# Association between Exercise and Blood Pressure in Hypertensive Residents: A Meta-Analysis

**DOI:** 10.1155/2022/2453805

**Published:** 2022-01-11

**Authors:** Zhu Zhu, Wu Yan, Qiurun Yu, Peihao Wu, Francis Manyori Bigambo, Jiaying Chen

**Affiliations:** ^1^Jiangsu Vocational Institute of Commerce, Nanjing 211168, China; ^2^School of Health Policy and Management, Institute of Healthy Jiangsu Development, Nanjing Medical University, Nanjing 211166, China; ^3^Department of Children Health Care, Nanjing Key Laboratory of Pediatrics, Children's Hospital of Nanjing Medical University, Nanjing 210008, China; ^4^School of Public Health, Nanjing Medical University, Nanjing 211166, China; ^5^Kangda College, Nanjing Medical University, 88 Chunhui Road, Lianyungang 222000, Jiangsu, China

## Abstract

**Background:**

Exercise is recommended as an effective lifestyle behaviour for adults to prevent and treat hypertension. In this study, a randomized-effect meta-analysis was used to analyse the influence of exercise interventions on blood pressure in patients with hypertension.

**Methods:**

Candidate papers were retrieved from PubMed, Web of Science, Embase, and Cochrane Library electronic databases, and 46 studies were finally included and analysed.

**Results:**

It was shown that preplanned walking (systolic blood pressure (SBP): WMD (weighted mean difference) = −5.94, 95% CI: −8.57, −3.30; diastolic blood pressure (DBP): WMD = −2.66, 95% CI: −3.66, −1.67), yoga (SBP: WMD = −5.09, 95% CI: −9.28, −0.89; DBP: WMD = −3.06, 95% CI: −5.16, −0.96), aquatic sports (SBP WMD = −7.53, 95% CI: −11.40, −3.65; DBP: WMD = −5.35, 95% CI: −9.00, −1.69), and football (SBP: WMD = −6.06, 95% CI: −9.30, −2.82; DBP: WMD = −5.55, 95% CI: −8.98, −2.13) had significant effects on blood pressure reduction. However, Tai Chi (SBP: WMD = −8.31, 95% CI: −20.39, 3.77; DBP: WMD = −3.05, 95% CI: −6.96, 0.87) and Qigong (SBP: WMD = −4.34, 95% CI: −13.5, 4.82; DBP: WMD = −3.44, 95% CI: −7.89, 1.01) did not significantly reduce blood pressure. The heterogeneity of the meta-analysis was high.

**Conclusion:**

Walking, yoga, aquatic sports, and football were feasible and independent lifestyle interventions, and they were effective options for treating hypertension. More scientifically designed randomized controlled trials are needed in the future to further compare different forms of exercise for the treatment of hypertension.

## 1. Background

With the rapid development of the social economy and the improvement of living standards, the prevalence of cardiovascular diseases is increasing. Hypertension is the most important risk factor for cardiovascular disease and should be given global attention. The World Health Organization (WHO) estimated that 25% of adults worldwide have hypertension, accounting for 4.5% of the global disease burden [1]. The number of adults with hypertension worldwide is predicted to increase to 1.56 billion by 2025, accounting for 29.2% of the total population [2]. Elevated blood pressure can lead to serious diseases such as coronary heart disease and heart failure.

Ruptured blood vessels caused by hypertension could be life-threatening [3]. The magnitude and healthcare burden of the condition highlight the need for effective strategies to prevent and manage hypertension. Despite the availability of effective antihypertensive drugs, the burden of hypertension is still increasing due to unhealthy behaviours, lack of screening awareness, and inadequate treatment and control [4, 5].

Exercise is a planned, structured, and repetitive activity carried out in leisure time to improve or maintain physical function. According to the WHO recommendations and guidelines for the diagnosis and treatment of hypertension in China (2019), increased aerobic exercise has been used as a supplement to antihypertensive drugs [6]. Evidence has shown that aerobic exercise of a certain intensity is helpful for the treatment of hypertension [7]. Exercise is one of the recommended lifestyle interventions for patients with cardiovascular disease, and it takes a variety of forms [8–10]. It is beneficial for hypertension patients to participate in moderate physical exercise to improve their heart function and strengthen their immunity. Patients could arrange to perform a reasonable amount of exercise based on age and blood pressure levels and choose appropriate forms of exercise, such as jogging, walking, and Tai Chi. Prospective epidemiological studies have shown that exercise is associated with lower blood pressure [11, 12]. Aerobic exercise also alleviates associated lipid abnormalities, which can also contribute to cardiovascular disease prevention [13].

Several cohort studies, randomized controlled trials (RCTs), and meta-analyses have explored the effects of aerobic or endurance exercise on blood pressure in patients with hypertension [14–16]. However, these types of studies did not evaluate the effects of different types of physical activity on blood pressure. Therefore, it is valuable to systematically evaluate the effect of each type of exercise on blood pressure control in patients with hypertension. We used published RCTs on the effects of exercise interventions on the blood pressure of hypertensive patients. Considering the number of available studies for each exercise, we finally determined six types of exercises, walking, yoga, aquatic sports, football, Tai Chi, and Qigong, for the meta-analysis. We evaluated the effects of each intervention to identify meaningful physical activity interventions for this special population.

## 2. Methods

### 2.1. Search Strategy

We searched the candidate articles in the PubMed, Web of Science, Embase, and Cochrane Library electronic databases for studies published before November 6, 2020. The following search terms were used in the databases:

1. “Hypertension” OR “Hypertensive” OR “High blood pressure” OR “Mean arterial” OR “Blood pressure” OR “Arterial pressure” OR “Systolic pressure” OR “Diastolic pressure” OR “Pulse pressure” OR “Venous pressure” OR “Pressure monitor” OR “Prehypertension” OR “BP”
2. “Tai Ji” OR “Tai-ji” OR “Tai Chi” OR “Tai Chiquan” OR “Qi gong” OR “Baduanjin” OR “Eight trigrams boxing” OR “Yoga” OR “Yogic” OR “Asana” OR “Pranayama” OR “Meditation” OR “Football” OR “Soccer” OR “Swim” OR “Natation” OR “Aquatic” OR “Brisk walking” OR “Walk” OR “Run”
3. “Randomized Controlled Trial” OR “Clinical Trial” OR “Randomized Clinical” OR “Random Allocation” OR “Randomization” OR “RCT”
4. #1 AND #2 AND #3

### 2.2. Study Selection and Data Extraction

Based on the PICOS principle of the Cochrane Handbook, the inclusion and exclusion criteria were determined, and the literature was strictly screened. The inclusion criteria of this meta-analysis were as follows: (1) The subjects were defined as patients with a diagnosis of hypertension. (2) The six types of exercise, walking, yoga, aquatic sports, football, Tai Chi, and Qigong, had corresponding controls. (3) Blood pressure values were reported after a prescribed period of exercise intervention. (4) The study reported the blood pressure and standard deviation of patients after the exercise intervention or changes in blood pressure and standard deviation before and after the intervention. (5) The study design was a randomized controlled trial. Two reviewers independently retrieved the papers and imported them to Endnote X9 to manage the search records. Screening of the titles and abstracts of the search records was conducted based on the inclusion criteria. The full texts of the eligible articles were also retrieved for further confirmation, and relevant data were extracted from the selected studies, including the first author name, types of exercise, country, number of participants, ages, sex, intervention, control, frequency, duration of the intervention, and outcome measures.

### 2.3. Bias Risk Assessment

The risk of bias in the included studies was assessed according to the Cochrane Handbook version 5.3.0. The risk of bias assessment was independently completed by two reviewers, and any conflict was resolved by a third reviewer.

### 2.4. Statistical Analysis

Since our data units were consistent, the combined results were expressed in weighted mean differences (WMDs) and 95% confidence intervals (CIs). There were two forms of mean blood pressure values, postintervention values and change values (the difference between postintervention values and baseline values). When the baseline blood pressure value was known, we tended to calculate the change because the change eliminated individual differences and was more comparable. *I*^2^ statistics were used to test the heterogeneity between the studies. An *I*^2^ ≥ 50% was considered to indicate high heterogeneity across the included studies, and the random-effects model was adopted for the corresponding effect value combination; otherwise, the fixed-effects model was adopted [17]. A positive value of the combined effect indicated that the mean blood pressure of the intervention group was higher than that of the control group, and a negative value indicated that the mean blood pressure of the intervention group was lower than that of the control group. Subgroup analysis was performed based on intervention duration to reduce heterogeneity. Sensitivity analyses were performed to assess the stability of the results, and funnel plots were used to further determine publication bias. We performed all the analyses with Stata 15.0, and a *P* < 0.05 was considered significant.

## 3. Results

### 3.1. Search Results

**Figure 1:**
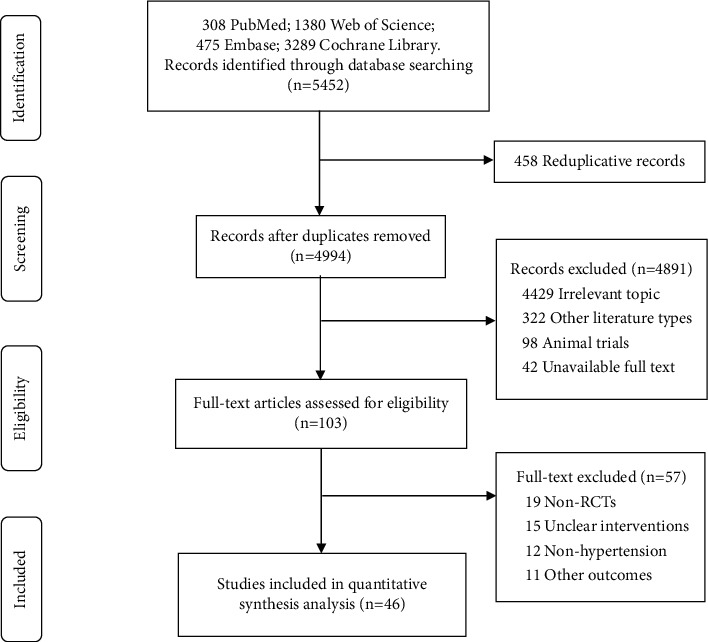
Flow diagram of literature screening.

A total of 5,452 candidate papers were initially identified. After reviewing the titles and abstracts, 103 studies were considered for further review. Of these studies, 15 were not RCT designs, 19 interventions were unclear, 12 studies were not conducted in a hypertensive population, and 11 focused on other outcome measures. Finally, 46 studies met the inclusion criteria for our meta-analysis. The detailed selection process is shown in Figure 1.

### 3.2. Characteristics of the Included Studies

A total of 3,058 hypertensive patients from 14 regions were included, and the sample size ranged from 18 to 770. The average age of the confirmed patients ranged from 37 years to 70.24 years. The duration of the interventions ranged from 2 weeks to 16 months. There were 11 studies that used walking as an intervention measure, which had the largest number of included studies, including 762 patients. There were 5 studies using football as an intervention, which had the least number of included studies, including 150 patients (79 in the intervention group and 71 in the control group). 25 studies used no treatments or no physical activity as control measures, while control groups in other studies received wellness education, standard lifestyle modifications, and standard clinical care. The characteristics of all included studies are shown in Table S1.

### 3.3. Quality of Included Studies

The quality assessment checklist was used to ensure that the included studies met the quality requirements. The results of the risk of bias assessment are shown in Table S2.

### 3.4. Meta-Analysis Results

#### 3.4.1. Walking

**Figure 2:**
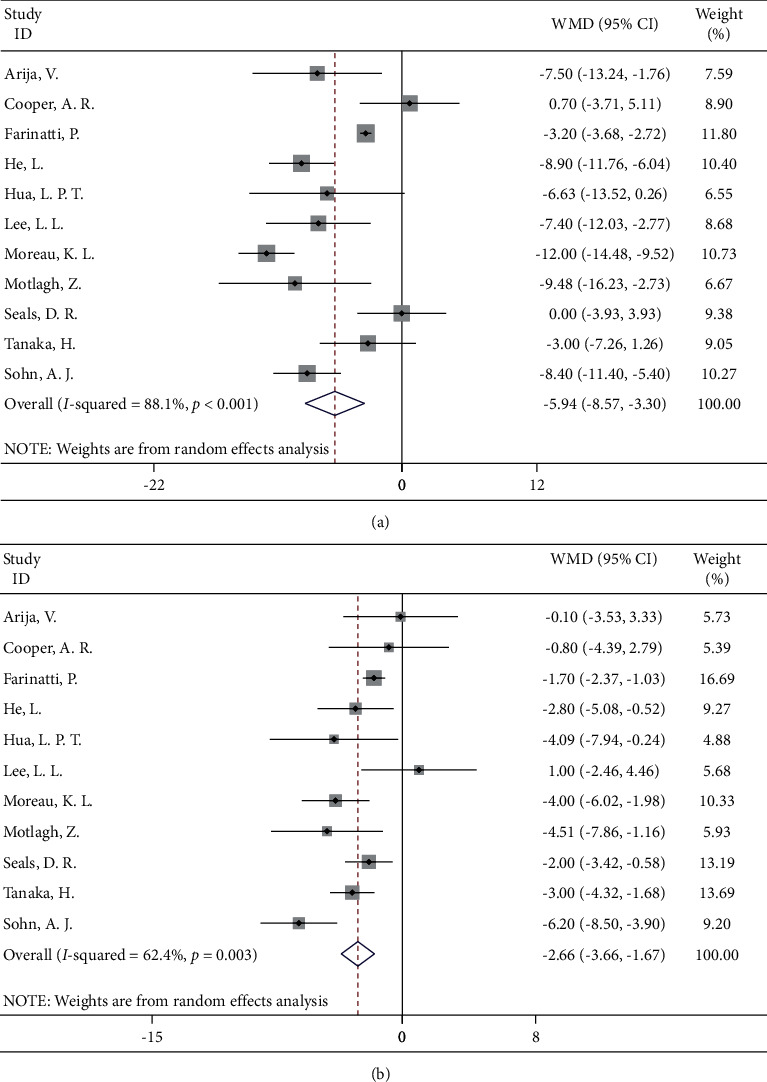
Forest map of walking and blood pressure: (a) systolic blood pressure; (b) diastolic blood pressure.

Eleven studies reported associations between walking and blood pressure changes, involving 762 hypertensive patients, with 442 in the experimental group and 320 in the control group. The pooled effect value showed that systolic blood pressure in the experimental group was reduced by 5.94 mmHg (95% CI: −8.57, −3.30) compared with the control group, and the difference was statistically significant. Similarly, the diastolic blood pressure of the walkers was 2.66 mmHg lower than that of the control group (95% CI: −3.66, −1.67) (Figure 2).

#### 3.4.2. Yoga

**Figure 3:**
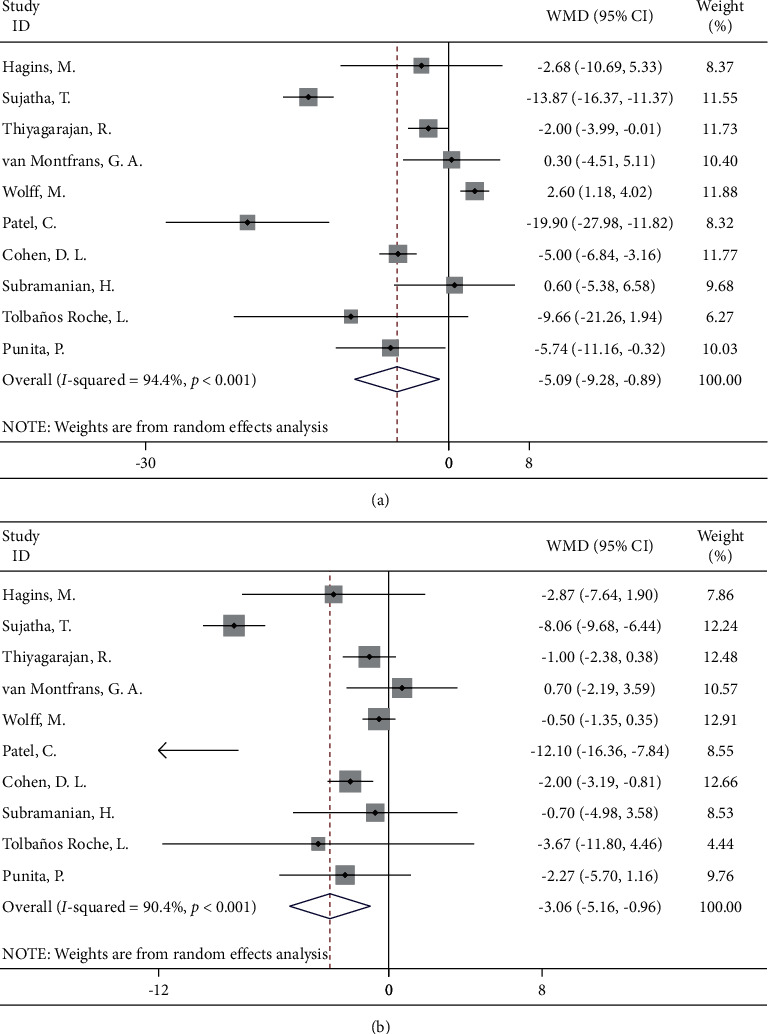
Forest map of yoga and blood pressure: (a) systolic blood pressure; (b) diastolic blood pressure.

Ten studies focused on the effect of a yoga intervention in hypertensive patients by randomized controlled trials. The results of the meta-analysis showed that, compared to the control group, hypertension patients who underwent the yoga intervention had significant benefits in lowering their blood pressure, with systolic blood pressure decreasing by 5.09 mmHg (95% CI: −9.28, −0.89) and diastolic blood pressure decreasing by 3.06 mmHg (95% CI: −5.16, −0.96) (Figure 3).

#### 3.4.3. Aquatic Sports

**Figure 4:**
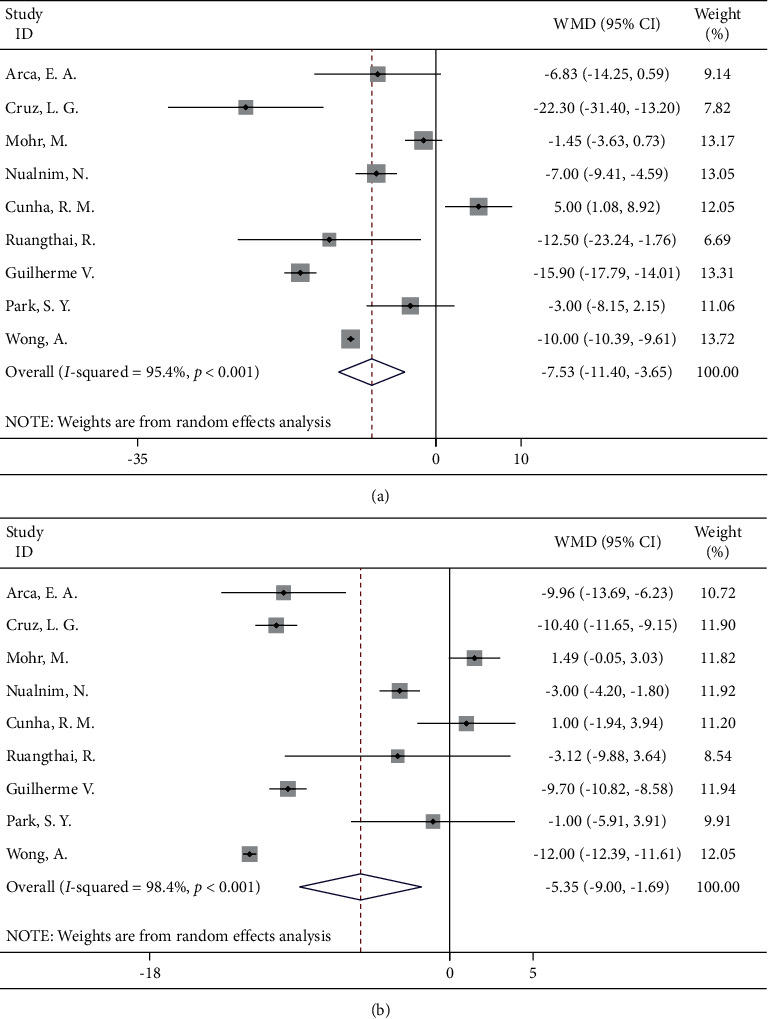
Forest map of aquatic sports and blood pressure: (a) systolic blood pressure; (b) diastolic blood pressure.

A total of nine studies compared the blood pressure of patients in the two groups after an aquatic sports intervention. A random-effects model was used for meta-analysis. The results showed that the improvement in systolic (WMD = −7.53, 95% CI: −11.40, −3.65) and diastolic (WMD = −5.35, 95% CI: −9.00, −1.69) blood pressure in the aquatic sports group was better than that in the routine nursing group, and the difference was statistically significant (Figure 4).

#### 3.4.4. Football

**Figure 5:**
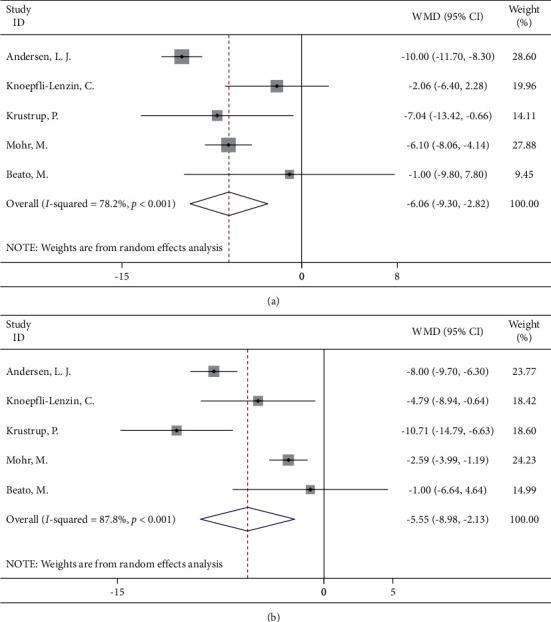
Forest map of football and blood pressure: (a) systolic blood pressure; (b) diastolic blood pressure.

Football is also one of the most common aerobic exercises, and five studies with football training interventions for people with high blood pressure were included, with three from the UK and the remaining two from Denmark and Sweden. Metaresults showed that the systolic blood pressure and diastolic blood pressure were significantly reduced in the patients with the soccer training intervention (WMD = −6.06, 95% CI: −9.30, −2.82; WMD = −5.55, 95% CI: −8.98, −2.13, respectively) (Figure 5).

#### 3.4.5. Tai Chi

**Figure 6:**
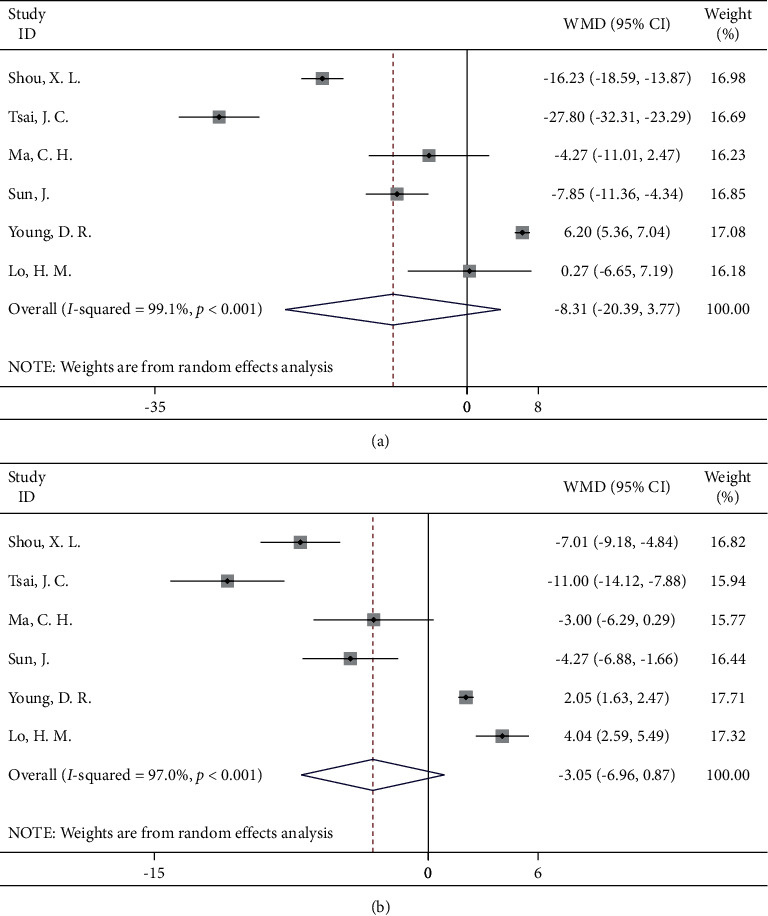
Forest map of Tai Chi and blood pressure: (a) systolic blood pressure; (b) diastolic blood pressure.

Six studies were included, and no significant effect of Tai Chi on blood pressure reduction was found for either systolic or diastolic blood pressure (WMD = −8.31, 95% CI: −20.39, 3.77; WMD = −3.05, 95% CI: −6.96, 0.87, respectively). However, we also found that there was a high degree of heterogeneity between the studies, which may be due to the large gaps in the sample size of each study, the length of the interventions, and the implementation of the intervention measures (Figure 6).

#### 3.4.6. Qigong

**Figure 7:**
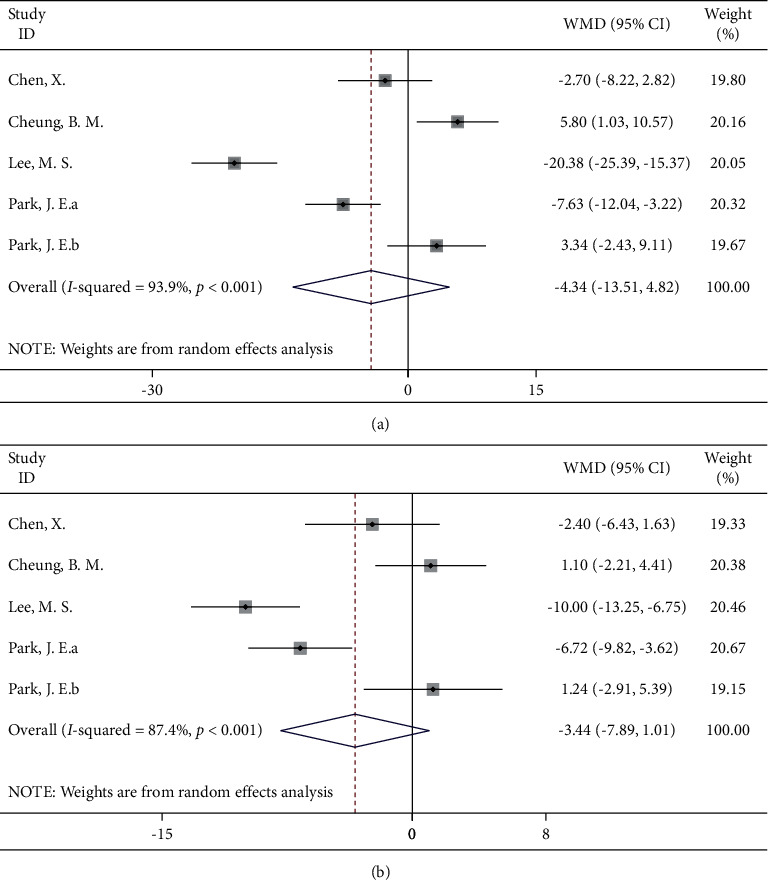
Forest map of Qigong and blood pressure: (a) systolic blood pressure; (b) diastolic blood pressure.

The five included studies included 131 patients receiving a Qigong intervention and 141 controls. The results of the meta-analysis showed that the blood pressure of the hypertensive patients receiving the Qigong intervention did not significantly decrease, which was consistent with the results of the Tai Chi intervention (Figure 7).

We performed a sensitivity analysis of the walking intervention training with the largest number of included studies, and the sensitivity analyses showed that the contribution of each study was balanced and that our results were robust **(**Figures S1 and S2). Based on the number of included papers, we tested the publication bias of “walking.” Egger's and Begg's tests showed no significant publication bias (Egger's test, *P*=0.755; Begg's test, *P*=0.124) (Figure S3). Funnel plots were drawn for all exercise types, and there was no apparent asymmetry in the funnel plot, indicating that the included papers did not have potential publication bias (Figure S4).

## 4. Discussion

In this study, we systematically reviewed the available evidence from the RCTs and conducted a meta-analysis to explore the effects of different types of exercise interventions on blood pressure improvement in patients with hypertension. Our results showed that walking, yoga, aquatic sports, and football had significant effects on blood pressure reduction. However, Tai Chi and Qigong did not significantly reduce blood pressure, and the results should be interpreted with caution because of the high heterogeneity of the meta-analysis.

Hypertension affects human health. There are many types of aerobic exercise, but which type is more effective and the underlying mechanisms remain to be explored. Our analysis showed that walking, yoga, aquatic sports, and football might lead to significant improvements in blood pressure in people with hypertension. Cohen et al. mentioned that yoga can promote mind-body integration and reduce stress, thus affecting blood pressure [18]. Some studies have shown that walking can reduce blood pressure, especially systolic blood pressure. In addition, recent studies have shown that walking can also reduce diastolic blood pressure. Furthermore, Farinatti et al. supposed that walking is a low-cost and flexible intervention that can be used as an alternative and complementary strategy for other sports [19]. He et al. proposed that brisk walking can effectively improve hypertension. Fast walking could reduce people's sedentary time, increase maximal oxygen uptake, decrease sympathetic excitation, and increase vagal excitation [20], thus reducing peripheral resistance and blood pressure. Next, Cohen et al. mentioned that yoga can promote mind-body integration and reduce stress, thus affecting blood pressure [18]. In addition, Sujatha et al. suggested that 12 weeks of yoga training can effectively reduce blood pressure and consequently reduce stress and anxiety [21], by reducing the excitement of the sympathetic nervous system and exciting the parasympathetic nervous system. Then, aquatic sports can also significantly improve hypertension. A study by Nualnim et al. found that aquatic sports can improve vascular compliance, resulting in endothelin-dependent vasodilation, and cause a decrease in systolic blood pressure [22]. Moreover, Cruz et al. revealed that aquatic sports training could normalize the level of refractory hypertension, which is related to the decrease in sympathetic excitation and the regulation of arterial baroreceptors [23, 24]. Last, Andersen et al. found that football training is effective in reducing blood pressure in middle-aged and elderly patients with mild-to-moderate hypertension [25]. Additionally, Mohr et al. proposed that 15 weeks of football training significantly reduced blood pressure [26]. Generally, walking, yoga, aquatic sports, and football have different mechanisms of blood pressure reduction. Walking, aquatic sports, and football can cause changes in nerve regulation and vasoactive substances through moderate-intensity aerobic exercise. Yoga may change blood pressure through emotional regulation.

Qigong has been used in the clinical setting for many years, but there is a dispute about whether it is effective. According to our findings, we cannot conclude that Qigong has positive therapeutic significance for patients with hypertension. Cheung et al. documented that the impact of Qigong on blood pressure is approximately 2 mmHg, and it has no clinical significance [27]. Furthermore, Park et al. also found that Qigong has no significant effect on hypertension [28]. In the same way, Tai Chi does not reach the medium intensity of exercise, so the effect is very small. Moreover, the design of many previous studies on these sports is not completely random, and there is a selection bias, which leads to serious differences in the results of many studies. Thus, more scientific and rigorous methods need to be designed to confirm whether Qigong and Tai Chi can control blood pressure.

Aerobic exercise is physical exercise of low to high intensity which depends primarily on the aerobic energy-generating process [29] such as jogging, walking, and swimming. Some research has indicated that aerobic exercise reduces blood pressure and improves ventricular functions. In addition, it is equally important that aerobic exercise can prevent brain degeneration and has short-term and long-term beneficial effects on psychological health [30].

Consequently, aerobic exercise was broadly recommended by various hypertension guidelines, such as Canada's 2018 hypertension guidelines [31], the Japanese Society of Hypertension Guidelines for the Management of Hypertension [32], and 2010 Chinese guidelines for the management of hypertension [33]. According to the WHO guidelines, hypertensive patients are encouraged to engage in aerobic exercise regularly for more than 30 minutes daily [29].

The proposed mechanisms of aerobic exercise on blood pressure control include neurohumoural, vascular, and structural adaptations [34]. Aerobic exercise increases endothelium-dependent vasodilation by increasing the production of nitric oxide (NO) [35, 36]. NO, a vital vasoactive molecule, normally dilates blood vessels and prevents platelets from getting sticky, which lowers blood pressure and protects the cardiovascular system [37, 38]. At the same time, oxidative stress is associated with the pathogenesis of hypertension [39, 40]. In hypertensive subjects, reactive oxygen species (ROS) were increased, which scavenge NO, thereby reducing NO bioavailability [38]. Consequently, decreased NO bioavailability is often present in hypertension and is the main characteristic of endothelial dysfunction. In brief, aerobic exercise training preserves vascular endothelial function by enhancing NO bioavailability and reducing oxidative stress [41].

Aerobic exercise can modulate the renin-angiotensin system (RAS), which is a dual system with two opposite arms [42]. The first arm is the classical arm formed by angiotensin converting enzyme (ACE), angiotensin type II (Ang II), and angiotensin type 1 receptors. The second arm is the counter regulatory arm consisting of ACE2 and Mas receptors [43]. In 2020, Magalhães found that aerobic exercise significantly increased urinary levels of ACE and plasma levels of ACE2, which was related to vasodilatation [44].

It is widely known that both heritable and lifestyle risk factors contribute to hypertension. In 2018, Evangelou reported that a total of 901 loci were associated with blood pressure [45], and the heritability of hypertension ranged from 30% to 60% [46]. At the same time, lifestyle modifications could offset the genetic risk of hypertension and lower blood pressure in participants with high risk. In addition to aerobic exercise, many lifestyle factors are associated with hypertension, including body mass index, diet, smoking, alcohol consumption, sedentary behaviours, and stress. In most populations, blood pressure increases linearly with increasing body mass index [47]. Dietary sodium is associated with elevated blood pressure, while dietary potassium lowers the risk of hypertension [48]. Regular frequent intake of fruits and vegetables is a protective factor against hypertension. Composite diets (such as the DASH diet, Mediterranean diet, and “prudent” diet) have been demonstrated to reduce the risk of hypertension [49]. In 2017, Kaiye Gao found that smoking was positively associated with the risk of hypertension, and the risk increased with age [50]. Regarding alcohol consumption, it was reported that any alcohol consumption was associated with an increase in the risk for hypertension in men. In women, there was no risk increase for the consumption of 1 to 2 drinks/day and an increased risk for higher consumption levels [51]. In 2020, a meta-analysis reported that high levels of total sedentary behaviour contributed to hypertension [52]. Meanwhile, psychological distress, acute panic disorder, and chronic depressive illness have been identified as dangerous risk factors for hypertension [53].

Our study has several strengths. First, to the best of our knowledge, this is the first systematic review of blood pressure changes in hypertensive patients with different exercise interventions. Second, many types of aerobic exercise and a large number of participants were included, which increased the accuracy of the effect estimate. Finally, the research design that we included was a randomized controlled trial, which had the highest level of evidence compared with other research design types, so the corresponding effect value combination results were more convincing. Some limitations of our study should be noted. First, our results showed a high heterogeneity level across the included studies, which may be due to the intervention periods and patients' ages. At the same time, the methodological differences in the included studies also contributed to the heterogeneity, and some studies did not blind the subjects and/or the outcome assessors, which may reduce the power of the results. In addition, due to the lack of a detailed description of exercise intensities and frequencies in the included studies, these key exercise principles were not taken into account in our analysis. Finally, the protocol in this study was not registered, and the evidence quality was not evaluated with the GRADE approach, which might affect the explanatory power of the results.

## 5. Conclusion

Our meta-analysis suggested that exercises such as walking, yoga, aquatic sports, football, Tai Chi, and Qigong were feasible and independent lifestyle interventions, and they were effective options for treating hypertension. Despite these encouraging findings, more scientifically designed randomized controlled trials are needed to further compare different forms of exercise for the treatment of hypertension to provide evidence-based recommendations for different types of exercise as antihypertensive treatments.

## Data Availability

The data used to support the findings of this study are available from the corresponding author upon request.

## Supplementary Materials

Supplementary Materials Table S1: basic characteristics of the studies. Table S2: summary of risk of bias assessment. Figure S1: sensitivity analysis of walking and systolic blood pressure. Figure S2: sensitivity analysis of walking and diastolic blood pressure. Figure S3: publication bias in studies on walking and systolic blood pressure. Figure S4: publication bias in studies on all exercise patterns and systolic blood pressure.
