# Supplementary material for: Association between Exercise and Blood Pressure in Hypertensive Residents: A Meta-Analysis
Source: Evid Based Complement Alternat Med. 2022 Jan 11;2022:2453805. doi: 10.1155/2022/2453805 (PMC8767394; doi:10.1155/2022/2453805)
Supplement: Supplementary Materials — Table S1: basic characteristics of the studies. Table S2: summary of risk of bias assessment. Figure S1: sensitivity analysis of walking and systolic blood pressure. Figure S2: sensitivity analysis of walking and diastolic blood pressure. Figure S3: publication bias in studies on walking and systolic blood pressure. Figure S4: publication bias in studies on all exercise patterns and systolic blood pressure. [file 2453805.f1.docx]

**Search strategy**

**Pubmed**

Search:((hypertension [Title/Abstract] OR hypertensive [Title/Abstract] OR "high blood pressure" [Title/Abstract] OR "mean arterial" [Title/Abstract] OR "blood pressure" [Title/Abstract] OR "arteria pressure" [Title/Abstract] OR "systolic pressure" [Title/Abstract] OR "diastolic pressure" [Title/Abstract] OR "pulse pressure" [Title/Abstract] OR "venouspressure" [Title/Abstract] OR "pressure monitor" [Title/Abstract] OR "prehypertension" [Title/Abstract] OR "BP" [Title/Abstract]) AND ("Tai Ji" [Title/Abstract] OR "Tai-ji"[Title/Abstract] OR "Tai Chi" [Title/Abstract] OR "Taijiquan” [Title/Abstract] OR "Qi gong" [Title/Abstract] OR "Baduanjin" [Title/Abstract] OR "Eight trigrams boxing" [Title/Abstract] OR yoga [Title/Abstract] OR yogic [Title/Abstract] OR asana [Title/Abstract] OR pranayama [Title/Abstract] OR meditation [Title/Abstract] OR "football" [Title/Abstract] OR "Soccer" [Title/Abstract] OR "swim" [Title/Abstract] OR "swimming" [Title/Abstract] OR "natation" [Title/Abstract] OR "aquatic" [Title/Abstract] OR "aquatic sports" [Title/Abstract] OR"brisk walking" [Title/Abstract] OR “walk” [Title/Abstract])) AND ("Randomized Controlled Trial" [Title/Abstract] OR "Clinical Trials Randomized" [Title/Abstract] OR "Trials, Randomized Clinical" [Title/Abstract] OR "Random Allocation" [Title/Abstract] OR "Allocation, Random" [Title/Abstract] OR "Randomization" [Title/Abstract] OR "Controlled Clinical Trials,Randomized" [Title/Abstract] OR "Clinical Trial" [Title/Abstract]) Sort by: Most Recent

**Web of science**

(hypertension OR hypertensive OR "high blood pressure" OR "mean arterial" OR "blood pressure" OR "arterial pressure" OR "systolic pressure" OR "diastolic pressure" OR "pulse pressure" OR "venous pressure" OR "pressure monitor" OR "prehypertension" OR "BP") AND ("Tai Ji" OR "Tai-ji" OR "Tai Chi" OR "Taijiquan" OR "Qi gong" OR "Baduanjin" OR "Eighttrigrams boxing" OR yoga OR yogic OR asana OR pranayama OR meditation OR "football" OR "Soccer" OR "swim" OR "swimming" OR "natation" OR "aquatic" OR "aquatic sports" OR "brisk walking" OR walk)AND ("Randomized Controlled Trial" OR "Clinical Trials, Randomized" OR "Trials, Randomized Clinical" OR "Random Allocation" OR "Allocation, Random" OR “Randomization" OR "Controlled Clinical Trials, Randomized" OR "Clinical Trial")

**Embase**

(hypertension:ab,ti OR hypertensive:ab,ti OR 'high blood pressure':ab,ti OR 'mean arterial':ab,ti OR 'blood pressure':ab,ti OR 'arterial pressure':ab,ti OR 'systolic pressure':ab,ti OR 'diastolic pressure':ab,ti OR 'pulse pressure':ab,ti OR 'venous pressure':ab,ti OR 'pressure monitor':ab,ti OR 'prehypertension':ab,ti OR 'bp':ab,ti) AND ('tai ji':ab,ti OR 'tai-ji':ab,ti OR 'tai chi':ab,ti OR 'taijiquan':ab,ti OR 'qi gong':ab,ti OR 'baduanjin':ab,ti OR 'eight trigrams boxing':ab,ti OR yoga:ab,ti OR yogic:ab,ti OR asana:ab,ti OR pranayama:ab,ti OR meditation:ab,ti OR 'football':ab,ti OR 'soccer':ab,ti OR 'swim':ab,ti OR 'swimming':ab,ti OR 'natation':ab,ti OR 'aquatic':ab,ti OR 'aquatic sports':ab,ti OR 'brisk walking':ab,ti OR walk:ab,ti) AND ('randomized controlled trial':ab,ti OR 'clinical trials, randomized':ab,ti OR 'trials, randomized clinical':ab,ti OR 'random allocation':ab,ti OR 'allocation, random':ab,ti OR 'randomization':ab,ti OR 'controlled clinical trials, randomized':ab,ti OR 'clinical trial':ab,ti)

**Cochrane:**

((hypertension [Title Abstract Keyword] OR hypertensive [Title Abstract Keyword] OR "high blood pressure" [Title Abstract Keyword] OR "mean arterial" [Title Abstract Keyword] OR "blood pressure" [Title Abstract Keyword] OR "arteria pressure" [Title Abstract Keyword] OR "systolic pressure" [Title Abstract Keyword] OR "diastolic pressure" [Title Abstract Keyword] OR "pulse pressure" [Title Abstract Keyword] OR "venouspressure" [Title Abstract Keyword] OR "pressure monitor" [Title Abstract Keyword] OR "prehypertension" [Title Abstract Keyword] OR "BP" [Title Abstract Keyword]) AND ("Tai Ji" [Title Abstract Keyword] OR "Tai-ji"[Title Abstract Keyword] OR "Tai Chi" [Title Abstract Keyword] OR "Taijiquan” [Title Abstract Keyword] OR "Qi gong" [Title Abstract Keyword] OR "Baduanjin" [Title Abstract Keyword] OR "Eight trigrams boxing" [Title Abstract Keyword] OR yoga [Title Abstract Keyword] OR yogic [Title Abstract Keyword] OR asana [Title Abstract Keyword] OR pranayama [Title Abstract Keyword] OR meditation [Title Abstract Keyword] OR "football" [Title Abstract Keyword] OR "Soccer" [Title Abstract Keyword] OR "swim" [Title Abstract Keyword] OR "swimming" [Title Abstract Keyword] OR "natation" [Title Abstract Keyword] OR "aquatic" [Title Abstract Keyword] OR "aquatic sports" [Title Abstract Keyword] OR"brisk walking" [Title Abstract Keyword] OR “walk” [Title Abstract Keyword])) AND ("Randomized Controlled Trial" [Title Abstract Keyword] OR "Clinical Trials Randomized" [Title Abstract Keyword] OR "Trials, Randomized Clinical" [Title Abstract Keyword] OR "Random Allocation" [Title Abstract Keyword] OR "Allocation, Random" [Title Abstract Keyword] OR "Randomization" [Title Abstract Keyword] OR "Controlled Clinical Trials,Randomized" [Title Abstract Keyword] OR "Clinical Trial" [Title Abstract Keyword])

**Table S1** Basic Characteristics of the Studies

| Exercise | Study (Year) | Country | Number of participants (T/C) | Finished number (T/C) | Participant age (years) | Sex  (male %) | Intervention | Control | Frequency | Duration |
| --- | --- | --- | --- | --- | --- | --- | --- | --- | --- | --- |
| Walking | Arija, V.(2018) | Spain | 237,175/62 | 207,152/55 | T:70.1±9.3  C:67.4±6.6 | T:27.3  C:23.7 | Walk and socio-cultural activities. | Standard clinical care | 120 minutes a week | 9 months |
|  | Cooper, A. R.(2000) | UK | 90,48/42 | 86,47/39 | T:46.2±9.4  C:49.4±8.9 | T:81.3  C:52.4 | Brisk walking | Notreatment | Morethan 30 minuteseveryday | 6weeks |
|  | Farinatti, P.(2016) | Brazil | 74,35/39 | 43,14/29 | — | — | Walk | Nophysical activity | Three times a week for 30 minutes | 16 months |
|  | He, L.(2018) | China | 46,23/23 | 42,20/22 | T:58±2  C:57±2 | T:0  C:0 | Brisk walking training | No treatments | Three times a week for 60 minutes | 12 weeks |
|  | Hua, L. P. T.(2009) | Canada | 20,10/10 | 20,10/10 | T:M:55.8±9.5  F:56.3±9.6  C:M:55.9±10.2 F:58.5±11.3 | T:50  C:50 | Walk | Maintain daily physical activities | Three times a week for 50-60 minutes | 12 weeks |
|  | Lee, L. L.(2007) | China | 202,102/100 | 184,91/93 | T:71.3±6.4  C:71.3±5.7 | T:62.7  C:54 | Community-based walking intervention | Usual primary health  care | — | 6 months |
|  | Moreau, K. L.(2001) | USA | 24,15/9 | 24,15/9 | T:53±2  C:55±1 | T:0  C:0 | 3-km walking | No treatment | Everyday | 24 weeks |
|  | Motlagh, Z.(2017) | Iran | 78,39/39 | 60,31/29 | T:54.05 ± 7.01  C:53.75 ± 8.27 | T:46.2 C:53.8 | Fast Walking | No treatment | Five times a week for 30 minutes at least | 3 months |
|  | Seals, D. R.(1991) | USA | 26,14/12 | 18,10/8 | T:63±2  C:61±3 | T:64.3  C:83.3 | Walk | No treatment | Three times a week for 30 minutes at least | 12 months |
|  | Tanaka, H.(1998) | USA | 22,11/11 | 22,11/11 | T:62.0±2.2  C:61.5±2.5 | T:63.6  C:81.8 | Walk | No treatment | Three or four times a week for 42±5 minutes | 6 months |
|  | Sohn, A. J.(2007) | USA | 18,8/10 | 18,8/10 | T:46.9±5.2  C:42.0±6.1 | T:25.0  C:40.0 | Walk | Wellness education | 5-7daysa weekfor 30 minutes | 6 months |
| Yoga | Hagins, M.(2014) | USA | 84,45/39 | 68,36/32 | T:56.4±9.78  C:52.45±12.19 | T:8.4  C:19.4 | Yoga | Active control (non-aerobic exercise) | Twice times a week for 55 minutes | 12 weeks |
|  | Sujatha, T.(2014) | China | 238,118/120 | 238,118/120 | 30-60 | T:46.6  C:45.8 | Yoga | No treatment | 5-dayintensive continuous training for 2 hour aday＋more than five time a week for 30-45 minutes | 12 weeks |
|  | Thiyagarajan, R.(2015) | India | 184,92/92 | 100,51/49 | T:44.08±9.42  C:42.47±9.00 | T:60.8  C:63.3 | Standard lifestyle modification＋Yoga | Standard lifestyle modification | Three times a week for 45 minutes | 12 weeks |
|  | van Montfrans, G. A.(1990) | Netherlands | 42,19/23 | 35,18/17 | T:40(24-56)  C:43(30-60) | T:55.6  C:47.1 | Hatha  yoga breathing and posture exercises | Relaxing in a  comfortable chair at home twice daily for 15 minutes | 1 hour a week under the guidance of a relaxation therapist＋twice a day at home  for 15 minutes | 8 weeks |
|  | Wolff, M.(2013) | Sweden | 65,28/27 | 65,28/27 | T:66.2±7.7  C:60.8±11.0 | T:67.9 C:59.3 | Yoga | No treatments | Once a week for 60 minutes with the guidance of an instructor＋once a day at home for 30 minutes | 12 weeks |
|  | Patel, C.(1975) | UK | 40,20/20 | 40,20/20 | —— | —— | Yoga | No treatments | —— | 3 months |
|  | Cohen, D.L.(2011) | USA | 78,46/32 | 43,12/31 | T:48.2 ± 1.6  C:48.3 ± 2.4 | T:50  C:50 | Iyengar yoga | Enhanced usual care | Twice a week for 70 minutes | 12 weeks |
|  | Subramanian, H.(2011) | India | 50,25/25 | 50,25/25 | T:23  C:23.32 | T:63.0  C:67.7 | Yoga | No treatments | More than 5 days a week for 30-45 minutes | 8 weeks |
|  | Tolbaños Roche, L.(2017) | Spain | 41,22/19 | 24,14/10 | T:54.14±9.65  C:54.40±8.92 | T:35.7  C:50.0 | Yoga | No treatments | 15 times in 12 months for 1 hour and 15 minutes | 2 months |
|  | Punita, P.(2015) | India | 80,40/40 | 55,30/25 | T:43.08±8.53  C:43.63±6.79 | T:76  C:83 | Yoga | No treatments | Three days a week for 45 minutes | 12 weeks |
| Aquatic sports | Arca, E. A. (2014) | Brazil | 33,19/14 | 33,19/14 | — | T:0  C:0 | Swim | No treatment | Three times a week for 50 minutes | 12 weeks |
|  | Cruz, L. G.(2017) | Brazil | 44,28/16 | 44,28/16 | T:54.4±1.2  C:52.4±1.5 | T:50.0  C:43.8 | Heated  water-based exercise | Habitual activities | Three times a week for 60 minutes | 12weeks |
|  | Mohr, M.(2014) | UK | 41,21/20 | 41,21/20 | T:44±2  C:45±2 | — | High-intensity intermittent swimming train | No treatment | Three times a week for 60 minutes | 15 weeks |
|  | Nualnim, M.(2012) | USA | 43,24/19 | 43,24/19 | T:58±2  C:61±2 | T:21.1  C:29.2 | Swim | No treatment | Threeorfour timesa week for 15-20 minutes | 12 weeks |
|  | Cunha, R. M.(2012) | Brazil | 16,16/16 | 16,16/16 | 66±2.94 | 0 | Exercise training  inside a pool with controlled temperature | Nophysical activity | 40minutes | —— |
|  | Ruangthai, R.(2020) | Thailand | 36,16/20 | 25,13/12 | T: 69.7 ± 6.5  C:66.7 ± 5.8 | T:7.7  C:41.7 | Exercise training  inside a pool with controlled temperature | Nophysical activity | Threetimesa week for 60 minutes | 12 weeks |
|  | Guilherme V.(2018) | Brazil | 32,16/16 | 32,16/16 | T:55.0 ± 5.9  C:52.4 ± 5.9 | T:50.0  C:62.5 | Exercise training  inside a pool with controlled temperature | Nophysical activity | Threetimesa week | 12 weeks |
|  | Park, S.Y.(2020) | South Korea | 53,28/25 | 53,28/25 | T:60.0 ± 10.0  C:60.0 ± 9.0 | T:0  C:0 | Heated-water exercise therapy | Land-based exercise | Four times a week for 60 minutes | 12 weeks |
|  | Wong, A.(2018) | South Korea | 100,52/48 | 100,52/48 | T:73 ± 4  C:74 ± 4 | T:0  C:0 | Swim | Regular level of physical activity | 25 to30 minutes a day, 3 to 4 days a week | 20 weeks |
| Football | Andersen, L. J. (2010) | Denmark | 25,15/10 | 22,13/9 | T:46.7±2.0  C:47.8±1.8 | T:100  C:100 | Football | Wellness education | Twice a week for 60 minutes | 3 months |
|  | Knoepfli-Lenzin, C. (2010) | Switzerland | 38,18/20 | 32,15/17 | T:37±4  C:38±5 | T:100  C:100 | Football | No treatment | Threetimesa week for 60 minutes | 12 weeks |
|  | Krustrup, P. (2013) | UK | 33,22/11 | 23,16/7 | 46(31-54) | T:100  C:100 | Football | Wellness education | Twice a week for 60 minutes | 6 months |
|  | Mohr, M. (2014) | UK | 41,21/20 | 40,20/20 | T:45±3  C:43±3 | T:0  C:0 | Football | No treatment | Threetimesa week for 60 minutes | 15 weeks |
|  | Beato, M.(2017) | UK | 24,10/14 | 24,10/14 | T: 42.9 ± 4.2  C:45.6 ± 4.8 | T:100  C:100 | Football | No treatment | 60 min per session | 12 weeks |
| Tai Chi | Shou, X. L.(2018) | China | 208,104/104 | 198,98/100 | T:M:52±6.46  F:51±7.09  C:M:52±8.98  F:51±7.54 | T:49.0  C:55.0 | General daily lifestyle intervention＋  24-Style Simplified taichi exercise | General daily lifestyle intervention | One to two times a day for 40–90 minutes | 3 months |
|  | Tsai, J. C.(2003) | China | 88,44/44 | 76,37/39 | T:51.66±16.3  C:50.56±9.8 | T: 51.4  C:48.7 | Taichi exercise | Maintainedusual lifestyle behaviors | Threetimesaweek for 50 minutes | 12 weeks |
|  | Ma, C. H.(2018) | China | 158,79/79 | 113,55/58 | T:70.24±10.25  C:69.71±10.84 | T:68  C:70 | 24-type Tai Chi +Well-  ness education | Wellness education | 3-5 times a week for 60 minutes | 6 months |
|  | Sun, J.(2015) | China | 300,150/150 | 266,136/130 | T:45-64:87(64%)  ≥65:49(36%)  C:45-64:90(70%)  ≥65:40(30%) | T:86  C:78 | Tai Chi  training | No-exercise-related activities | 3 hours a week and 2-hour practice by themselves  at home | 12 months |
|  | Young, D. R.(1999) | South Korea | 62,31/31 | 62,31/31 | T:67.0±7.9  C:66.4±5.1 | T:80.7  C:77.4 | Tai Chi  training | No treatment | 4 to 5 days  per week and 30 to 45 minutes per day | 12 weeks |
|  | Lo, H. M.(2012) | China | 74 | 58,27/31 | 58.47±7.46 | 56.8 | Tai Chi  training | Routine care | Three times a week for 60 minutes | 8 weeks |
| Qigong | Chen, X.(2016) | Malaysia | 76,45/33 | 46,21/25 | T:49.7±6.08  C:47.5±5.68 | T:33.3  C:28.0 | One-minute qigong exercise | One-minute reading session | Five times a week for 1 minutes | 2 weeks |
|  | Cheung, B. M. (2015) | China | 91,47/44 | 89,47/41 | T:57.2±9.5  C:51.2±7.4 | T:44.7  C:39.0 | Guolin qigong | Conventional exercise | 60 minutes in morning and 15 minutes at night every day | 16 weeks |
|  | Lee, M. S. (2003) | South Korea | 65,33/32 | 58,29/29 | T:55.8±96.2  C:57.1±7.6 | T:34.5  C:44.8 | Shuxinpingxue gong | No treatment | Twice a week for 120 minutes | 8 weeks |
|  | Park, J. E.a. (2012) | South Korea | 40,19/21 | 40,19/21 | T:52.0 (43.0, 61.0)  C:54.0 (45.0, 62.0) | T:68.4  C:61.9 | Qigong | No treatment | Threetimes per week for30minutesinclassandmore than 2 times per week at home | 8 weeks |
|  | Park, J. E.b. (2017) | South Korea | 61,29/32 | 52,25/27 | T:54.52±6.96  C:5.2.93±8.45 | T:52  C:81.5 | Dongeui Qigong | No treatment | More than five times aweek for 50 minutes | 12 weeks |

**Table S2** Summary of risk of bias assessment

| Study | Random sequence generation | Allocation concealment | Blinding of participants and personnel | Incomplete outcome data | Selective reporting | Other bias |
| --- | --- | --- | --- | --- | --- | --- |
| Arija, V. (2018) | Unclear | Unclear | Low | Low | Unclear | Unclear |
| Cooper, A. R. (2000) | Low | Low | Low | Low | Unclear | Unclear |
| Farinatti, P. (2016) | Unclear | Unclear | Low | Low | Unclear | Unclear |
| He, L. (2018) | Unclear | Unclear | Low | Low | Unclear | Low |
| Hua, L. P. T. (2009) | Low | Unclear | Low | Low | Unclear | Unclear |
| Lee, L. L. (2007) | Low | Low | Low | Low | Unclear | Unclear |
| Moreau, K. L. (2001) | High | High | Low | Low | Unclear | Unclear |
| Motlagh, Z. (2017) | Low | Low | Low | Low | Low | Low |
| Seals, D. R. (1991) | High | High | Low | Low | Low | Unclear |
| Tanaka, H. (1998) | High | Low | Low | Low | Low | Low |
| Sohn, A. J. (2007) | Low | Unclear | Low | Low | Low | Unclear |
| Hagins, M. (2014) | Low | Low | Low | Low | Low | Unclear |
| Sujatha, T. (2014) | High | High | Low | Low | Low | Unclear |
| Thiyagarajan, R. (2015) | Unclear | High | High | Low | Unclear | Unclear |
| van Montfrans, G. A. (1990) | Low | Low | Low | Low | Unclear | Unclear |
| Wolff, M. (2013) | Low | High | Low | Low | Unclear | Unclear |
| Patel, C. (1975) | Unclear | Unclear | Low | Low | Unclear | Unclear |
| Cohen, D.L. (2011) | Unclear | Unclear | Low | Low | Low | Unclear |
| Subramanian, H. (2011) | Low | Unclear | Low | Low | Low | Low |
| Tolbaños Roche, L. (2017) | Low | Low | Low | Low | Unclear | Unclear |
| Punita, P. (2015) | Low | Low | Low | Low | Unclear | Unclear |
| Arca, E. A. (2014) | Unclear | Unclear | Low | Low | Low | Unclear |
| Cruz, L. G. (2017) | Low | Low | Low | Low | Unclear | Low |
| Mohr, M. (2014) | Unclear | Unclear | Low | Low | Unclear | Low |
| Nualnim, M. (2012) | Unclear | Unclear | Low | Low | Unclear | Low |
| Cunha, R. M. (2012) | Unclear | High | Low | Low | Unclear | Unclear |
| Ruangthai, R. (2020) | Low | Low | Low | Low | Unclear | Unclear |
| Guilherme V. (2018) | Low | Low | Low | Low | Unclear | Low |
| Park, S.Y. (2020) | Low | Unclear | Low | Low | Unclear | Unclear |
| Wong, A. (2018) | Low | Low | Low | Low | Low | Unclear |
| Andersen, L. J. (2010) | Unclear | Unclear | Low | Low | Low | Low |
| Knoepfli-Lenzin, C. (2010) | Unclear | Unclear | Low | Low | Unclear | Unclear |
| Krustrup, P. (2013) | Unclear | Unclear | Low | Low | Unclear | Unclear |
| Mohr, M. (2014) | Unclear | Unclear | Low | Low | Unclear | Unclear |
| Beato, M. (2017) | Low | Unclear | Low | Low | Low | Low |
| Shou, X. L. (2018) | Unclear | Unclear | Low | Low | Unclear | Unclear |
| Tsai, J. C. (2003) | Unclear | Unclear | Low | Low | Unclear | Unclear |
| Ma, C. H. (2018) | Low | Low | Low | Low | Unclear | Low |
| Sun, J. (2015) | Unclear | Unclear | Low | Low | Low | Unclear |
| Young, D. R. (1999) | Low | Low | Low | Low | Low | Unclear |
| Lo, H. M. (2012) | High | High | Low | Low | Low | Unclear |
| Chen, X. (2016) | Low | High | Low | Low | Unclear | Unclear |
| Cheung, B. M. (2015) | Low | Unclear | Low | Low | Unclear | Unclear |
| Lee, M. S. (2003) | Unclear | Unclear | Low | Low | Unclear | Unclear |
| Park, J. E.a. (2012) | Low | Low | Low | Low | Low | Unclear |
| Park, J. E.b. (2017) | Low | Low | Low | Low | Low | Low |


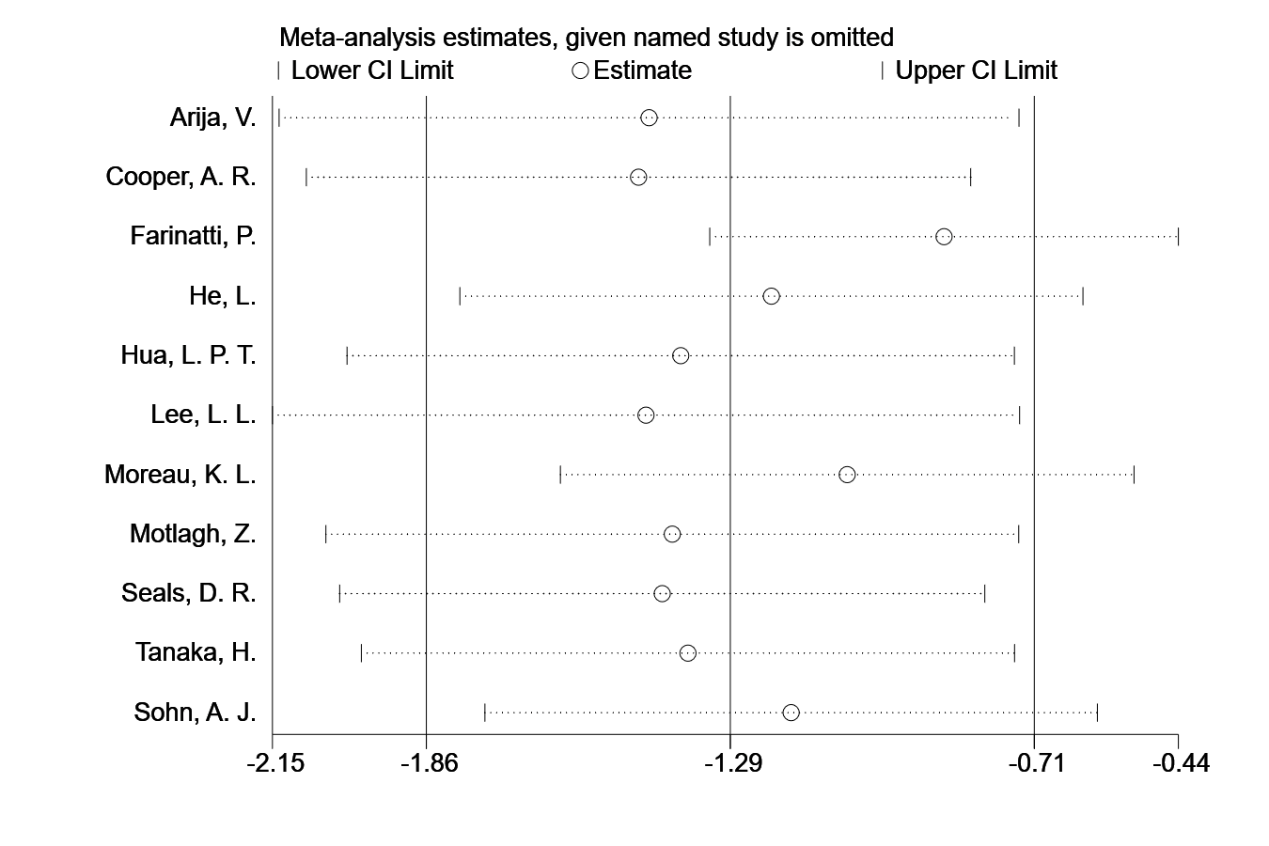
**Figure S1**. Sensitivity analysis of walking and systolic blood pressure


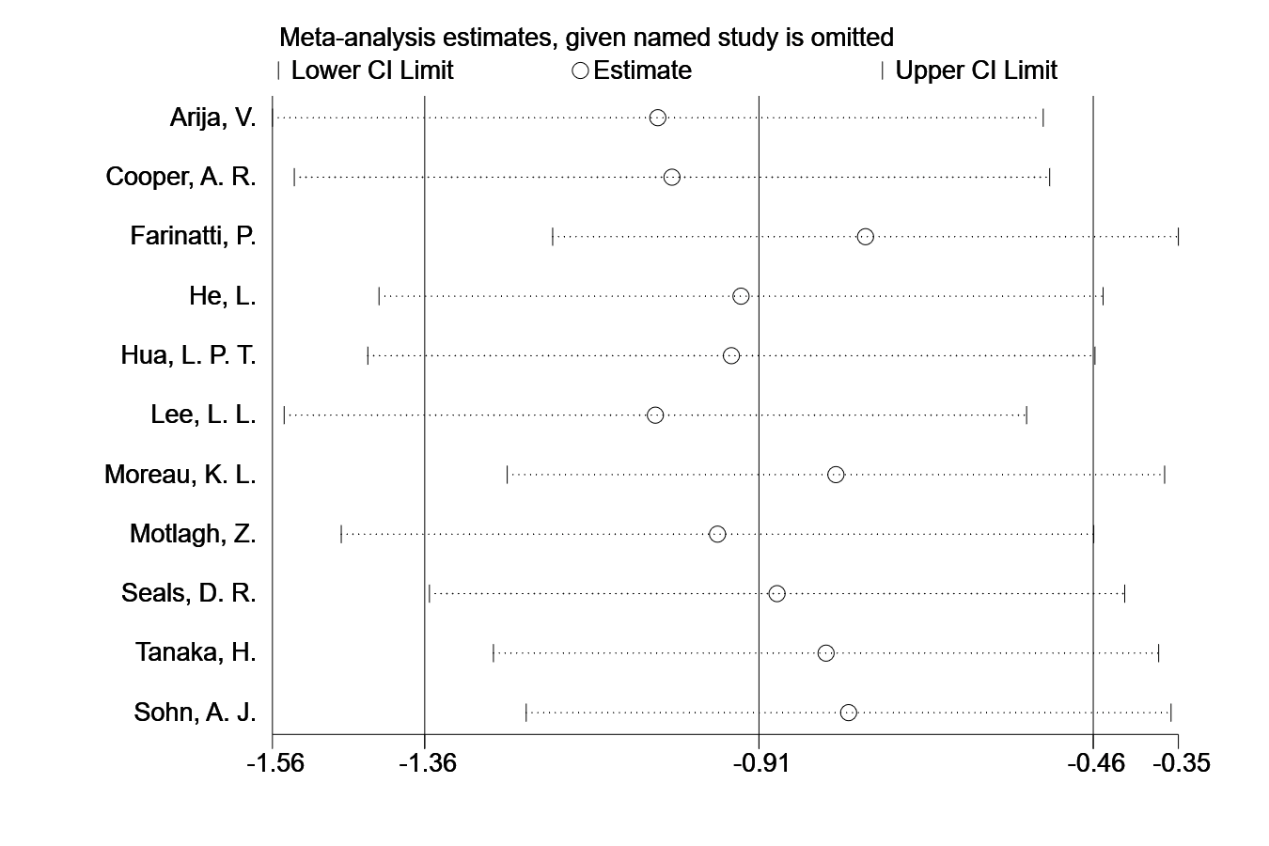


**Figure S2**. Sensitivity analysis of walking anddiastolic blood pressure

**Figure S3**. Publication bias in studies on walking and systolic blood pressure


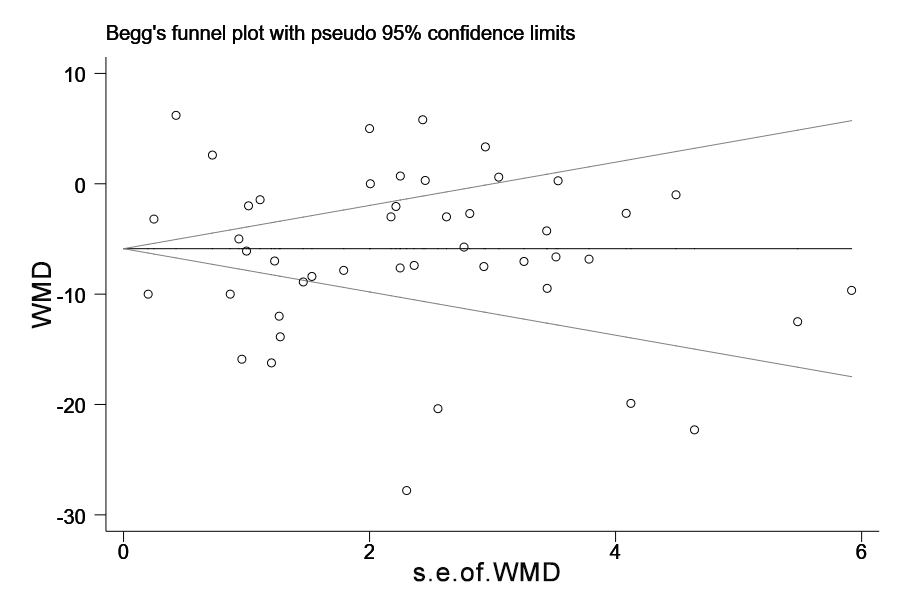


**Figure S4**. Publication bias in studies on all exercise patterns and systolic blood pressure
